# Relationships Between Chemoreflex Responses, Sleep Quality, and Hematocrit in Andean Men and Women

**DOI:** 10.3389/fphys.2020.00437

**Published:** 2020-05-06

**Authors:** Erica C. Heinrich, Jeremy E. Orr, Dillon Gilbertson, Cecilia Anza-Ramirez, Pamela N. DeYoung, Matea A. Djokic, Noemi Corante, Gustavo Vizcardo-Galindo, Jose L. Macarlupu, Eduardo Gaio, Frank L. Powell, Atul Malhotra, Francisco C. Villafuerte, Tatum S. Simonson

**Affiliations:** ^1^Division of Pulmonary, Critical Care, and Sleep Medicine, Department of Medicine, University of California, San Diego, San Diego, CA, United States; ^2^Laboratorio de Fisiología Comparada/Fisiología del Transporte de Oxígen, Facultad de Ciencias y Filosofía, Universidad Peruana Cayetano Heredia, Lima, Peru; ^3^Faculty of Medicine, University of Brasília, Brasília, Brazil

**Keywords:** hypoxia, sleep, control of breathing, excessive erythrocytosis, high altitude

## Abstract

Andean highlanders are challenged by chronic hypoxia and many exhibit elevated hematocrit (Hct) and blunted ventilation compared to other high-altitude populations. While many Andeans develop Chronic Mountain Sickness (CMS) and excessive erythrocytosis, Hct varies markedly within Andean men and women and may be driven by individual differences in ventilatory control and/or sleep events which exacerbate hypoxemia. To test this hypothesis, we quantified relationships between resting ventilation and ventilatory chemoreflexes, sleep desaturation, breathing disturbance, and Hct in Andean men and women. Ventilatory measures were made in 109 individuals (*n* = 63 men; *n* = 46 women), and sleep measures in 45 of these participants (*n* = 22 men; *n* = 23 women). In both men and women, high Hct was associated with low daytime SpO_2_ (*p* < 0.001 and *p* < 0.002, respectively) and decreased sleep SpO_2_ (mean, nadir, and time <80%; all *p* < 0.02). In men, high Hct was also associated with increased end-tidal P_CO2_ (*p* < 0.009). While ventilatory responses to hypoxia and hypercapnia did not predict Hct, decreased hypoxic ventilatory responses were associated with lower daytime SpO_2_ in men (*p* < 0.01) and women (*p* < 0.009) and with lower nadir sleep SpO_2_ in women (*p* < 0.02). Decreased ventilatory responses to CO_2_ were associated with more time below 80% SpO_2_ during sleep in men (*p* < 0.05). The obstructive apnea index and apnea-hypopnea index also predicted Hct and CMS scores in men after accounting for age, BMI, and SpO_2_ during sleep. Finally, heart rate response to hypoxia was lower in men with higher Hct (*p* < 0.0001). These data support the idea that hypoventilation and decreased ventilatory sensitivity to hypoxia are associated with decreased day time and nighttime SpO_2_ levels that may exacerbate the stimulus for erythropoiesis in Andean men and women. However, interventional and longitudinal studies are required to establish the causal relationships between these associations.

## Introduction

Chronic Mountain Sickness (CMS) in native and long-term high-altitude residents is characterized by excessive erythrocytosis and results in severe hypoxemia, sleep disturbance, and neurological symptoms (León-Velarde et al., 2005; Villafuerte and Corante, 2016). In the southern Andes of Peru at 3,825 m, the estimated average prevalence of excessive erythrocytosis is 6% of adult men and 3% of adult women; while in the central Andes at 4,340 m, the prevalence increases to 25 and 15%, respectively (Monge et al., 1989, 1992; De Ferrari et al., 2014). Age is a compounding risk factor whereby the prevalence of excessive erythrocytosis is >30% in highlanders by their mid-50s in both sexes above 4,000 m, including about 77% of post-menopausal women (Monge et al., 1989, 1992; León-Velarde et al., 1997).

Individuals with excessive erythrocytosis are at higher risk of pulmonary hypertension and adverse cardiovascular events (Penaloza et al., 1971; Penaloza and Arias-Stella, 2007; Corante et al., 2018). While CMS and excessive erythrocytosis are highly prevalent in Andeans (Villafuerte and Corante, 2016), these conditions are rare among Tibetans who tend to exhibit sea-level hemoglobin concentration despite residence at comparable altitudes (Wu et al., 2005). The range of hematocrit (Hct) is notable among Andeans (Beall et al., 1998), and individual variation in traits that affect oxygen (O_2_) delivery, including ventilatory control and its impact on sleep disordered breathing, may contribute to the excessive production of red blood cells.

Andeans also demonstrate notable hypoventilation and a lower average hypoxic ventilatory response (HVR) compared to Tibetans (Zhuang et al., 1993; Beall et al., 1997). While acute HVR appears to be reduced in Andeans with or without CMS, lower ventilatory sensitivities to CO_2_ have been observed specifically in individuals with CMS compared to healthy Andean controls (Fatemian et al., 2003; Leon-Velarde et al., 2003). Lower central and peripheral chemoreflex set points might lead CMS individuals to hypoventilate at a given CO_2_ partial pressure (P_CO2_), resulting in lower arterial O_2_ saturation and increased erythropoietic responses.

Ventilatory sensitivities to O_2_ and CO_2_ also play a key role in sleep disordered breathing in highlanders (Spicuzza et al., 2004; Julian et al., 2013). High ventilatory sensitivity to hypoxia can lead to Cheyne-Stokes respiration (periodic breathing, waxing, and waning breathing patterns) at high altitude (Lahiri et al., 1983; Masuyama et al., 1989; Goldenberg et al., 1992; Küpper et al., 2008), while low ventilatory sensitivity to hypoxia or CO_2_ can lead to more severe desaturation during sleep and/or prolonged desaturation periods (Azarbarzin et al., 2019). Both situations can lead to maladaptive cardiovascular outcomes. Sleep disordered breathing is more prevalent in Peruvian highlanders than lowlanders at sea level (Pham et al., 2017a), and nocturnal hypoxemia and sleep apnea events are separately associated with excessive erythrocytosis and glucose intolerance, respectively (Pham et al., 2017b). The severity, frequency, and duration of intermittent desaturation on top of chronic hypoxemia likely influence erythropoiesis and other hypoxia-related pathways that contribute to negative cardiovascular outcomes.

We aimed to determine whether higher Hct is associated with lower ventilatory chemosensitivity and/or more frequent or severe desaturation events during sleep, and tested whether the uniquely low chemoreflex sensitivities observed in this population may contribute to more severe desaturation during sleep. By examining Hct, ventilatory chemoreflexes, and sleep quality in the same individuals, we identified, for the first time, associations between individual ventilatory control and sleep disturbance profiles that are linked to Hct in both men and women. We also show decreased heart rate response (HRR) to hypoxia in men with high Hct. These findings highlight relevant individual and sex-specific profiles that should be prioritized for functional investigation in future studies.

## Materials and Methods

### Ethical Approval

This study was conducted in accordance with the Declaration of Helsinki, except for registration in a database, and was approved by the University of California, San Diego Human Research Protection Program. Participants provided written consent in their native language (Spanish).

### Participants and Preliminary Screening Visit

This study took place at the Instituto de Investigaciones de la Altura laboratory in the city of Cerro de Pasco, Peru (∼4,340 m; population ∼70,000). Men and women who were lifelong residents of Cerro de Pasco were recruited by word of mouth and flyers. Inclusion criteria was defined as individuals 18 to 65 years old with at least three previous generations of self-reported high-altitude (>2,500 m) Andean ancestry (self-identified ancestry and geographical location of their parents and grandparents). Women of reproductive age completed a urine pregnancy test to verify they were not pregnant. Participants were excluded if they had a self-reported history of pulmonary, cardiovascular, or renal disease to rule out secondary CMS cases (Villafuerte and Corante, 2016). Participants were also excluded if they were current smokers or regular drinkers, had recently undergone blood transfusions or phlebotomies, had traveled to low-altitude (<4,000 m) during the previous six months, or demonstrated abnormal cardiac or pulmonary function during screening procedures (EKG and spirometry). Table 1 provides an overview of the study population demographics.

**TABLE 1:** Participant demographics for total study population.

Variable	Men (*n* = 63)	Women (*n* = 46)	*p*-value
Age (y)	43.0 ± 12.9	40.1 ± 13.5	0.254
BMI (kg/m^2^)	25.7 ± 3.2	27.7 ± 4.4	0.008
CMS Score	4.8 ± 5.3	1.5 ± 2.1	<0.001
SBP (mmHg)	114.3 ± 15.4	110.5 ± 14.8	0.192
DBP (mmHg)	76.4 ± 11.4	74.8 ± 10.3	0.433
Hct (%)	59.7 ± 8.1	50.5 ± 6.4	<0.001
SpO_2_ (%)	83.4 ± 5.8	84.5 ± 4.8	0.250
PET_CO2_ (mmHg)	29.4 ± 4.5	28.8 ± 2.6	0.104
HR (bpm)	73.7 ± 11.8	73.8 ± 10.5	0.949

*Values listed as mean ± standard deviation and represent measures taken during wakefulness. *p*-values represent comparisons across sex groups.*

Participants were asked not to drink alcohol or caffeine, nor consume/chew coca leaves or tea, for 8 h prior to testing and were asked to refrain from taking anti-inflammatory drugs for 24 h prior to testing. Medical histories and physical examinations were performed during a preliminary screening session to verify no prior history of cardiovascular or pulmonary disease or current use of interfering medications that would preclude ventilatory control assessment. During this visit, participants were assessed for presence and severity of CMS based on the Qinghai CMS scoring criteria, which considers hemoglobin concentration at the altitude of residence and the presence and severity of the following symptoms: breathlessness and/or palpitations, sleep disturbance, cyanosis, dilation of veins, paresthesia, headache, and tinnitus (Wu et al., 1997, 1998; León-Velarde et al., 2005).

Average Hct was determined from duplicate microcentrifuged blood samples obtained from a single fingertip capillary blood draw. Individuals were determined to have excessive erythrocytosis if they had an Hct ≥ 63 % for men or ≥57% for women, which are equivalent to the thresholds for hemoglobin in the Qinghai CMS Score criteria (Wu et al., 1997, 1998; León-Velarde et al., 2005). Our group has previously determined that mean corpuscular hemoglobin concentration is effectively similar between men with Hct ≤ 54% (*n* = 84, MCHC = 34.1 ± 1.8 g/dl) and Hct ≥ 63% (*n* = 95, MCHC = 33.0 ± 1.7 g/dl). For this, and practical reasons, we only measured Hct in this study.

Ventilatory control was measured in 109 individuals [*n* = 63 men and 46 women (31 pre and 15 post menopause)] and sleep parameters in a subset of this group [*n* = 45; 22 men and 23 women (14 pre and 9 post menopause)].

### Ventilatory Chemoreflex Measurements

Participants completed a 10-min abbreviated version of the ventilatory response protocol to allow acclimation to the devices and determine appropriate individual gas flows to reach their target SpO_2_ and end-tidal P_CO2_ (PET_CO2_) values. Participants returned after a >30-min rest period to complete the full protocol. This rest period and preparation for the next test allowed sufficient time for recovery from hypoxic ventilatory decline induced by the screening session hypoxia exposure (Easton et al., 1988; Robbins, 2007).

**FIGURE 1:**
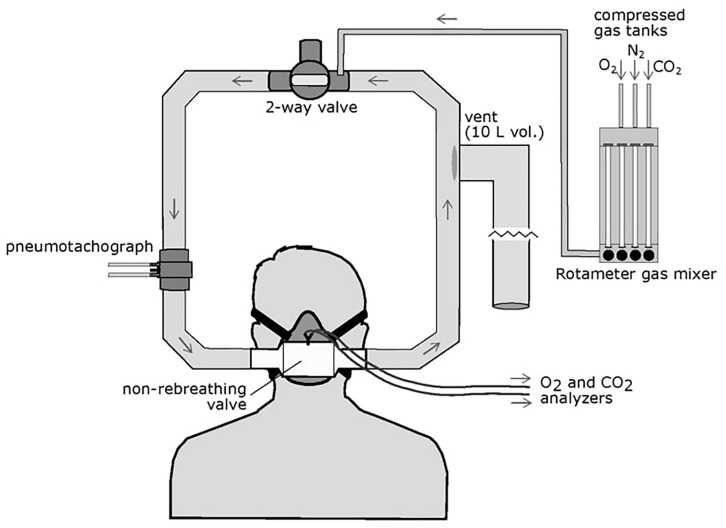
Schematic of the HVR experimental setup. The participant wore an oral-nasal mask attached to a non-rebreathing valve which allowed one-way airflow through the circuit. A two-way valve can be opened to allow entry or room air of closed to allow flow of mixed gases through the circuit. Gas mixtures are controlled *via* a rotameter attached to compressed O_2_, N_2_, and CO_2_ gas tanks and delivered to the breathing circuit. Inspired flow is measured by a pneumotachograph upstream of the mask. A large volume (10 L), low resistance vent is located downstream of the non-rebreathing valve to prevent pressure build up in the circuit but did not allow room air to enter the circuit.

We used a protocol developed over several years for measuring the steady-state isocapnic HVR. This method is a modification of the protocol utilized by the Severinghaus laboratory (Sato et al., 1992, 1994) as previously described (Hupperets et al., 2004; Basaran et al., 2016) (Figure 1 illustrates the experimental setup). During testing, participants sat in a chair in a semi-recumbent position and a mask was placed over the mouth and nose (7600 V2 Oro-Nasal Mask, Hans Rudolph Inc., Shawnee, KS, United States); leaks were checked by having the participant inhale against a closed inspiratory valve to ensure a vacuum was produced. The mask was connected to a one-way, vented partial rebreathing circuit with a non-rebreathing valve (2700 Series, Large, Hans Rudolph Inc.). A three-way valve upstream of the mask allowed either room air or N_2_/O_2_/CO_2_ gas mixtures to flow into the circuit. O_2_ and CO_2_ were continuously sampled from the non-rebreathing valve directly in front of the mouth by CO_2_ (Model 17515A, VacuMed, Ventura, CA, United States) and O_2_ (Model 17620, VacuMed) analyzers which pulled gas at a rate of 200 ml/min.

Gas analyzers were calibrated daily using a compressed gas mixture with 4% CO_2_, 16% O_2_, and nitrogen balance (Airgas, Radnor, PA, United States). Inspiratory flow was measured by a Fleisch pneumotachograph upstream of the non-rebreathing valve and mask and connected to a carrier demodulator (model CD15, Validyne, Northridge, CA, United States). SpO_2_ and heart rate were measured by a pulse oximeter (Nellcor model N395, Medtronic, Minneapolis, MN, United States) with a surface probe placed on the forehead. All analog signals were processed through a PowerLab 8/30 (ADInstruments, Colorado Springs, CO, United States) and sent digitally to a laptop computer (HP Probook, HP Inc., Palo Alto, CA, United States). Raw data was recorded in LabChart 8 (ADInstruments, Colorado Springs, CO, United States).

Gas mixtures were manually controlled with a three-channel rotameter flow meter (Matheson Gas Products, Montgomeryville, PA, United States) that delivered mixtures upstream of the mask at flow rates sufficient to prevent rebreathing. Participants breathed ambient air for 5 min followed by 10 min of mild hyperoxia (simulating 30–40% F_I_O_2_ at sea level). This hyperoxic phase was intended to reverse hypoxic ventilatory decline in these participants due to continuous hypoxemia at high-altitude (Pamenter and Powell, 2016). To determine the HVR, participants then breathed a normoxic gas mixture (159 mmHg PO_2_, simulating 21% F_I_O_2_ at sea-level and increasing SpO_2_ above resting levels during room air breathing) for 5 min followed by 5 min of hypoxia during which we targeted 3 min of stable SpO_2_ between 80 and 85%. While this SpO_2_ level is near the resting value for this group, to determine the HVR, ventilation at this treatment level was compared to the “sea-level” experimental condition at which saturation levels were 97.3 ± 2.4% across all subjects. Isocapnia was achieved throughout the hypoxic phase by manually adding CO_2_ to maintain the average PET_CO2_ value from the last 1 min of the normoxic period. To determine ventilatory responses to CO_2_ (hypercapnic ventilatory response, HCVR) and a combined hypoxic and hypercapnic stimulus (hypercapnic HVR), participants then breathed normoxic air (159 mmHg PO_2_) with PET_CO2_ 5 mmHg higher than their previous isocapnic value for 5 min. This was followed by 5 min of isocapnic hypoxia in which SpO_2_ was stable at a value between 80 and 85%. Target PET_CO2_ values were maintained within 1 mmHg of the target isocapnic value.

The HVR and hypercapnic HVR were calculated as the change in ventilation per decrease in SpO_2_. HCVR was calculated as the change in ventilation per mmHg increase in PET_CO2_. The HRR to hypoxia was calculated as the change in heart rate per decrease in SpO_2_ during the HVR treatment steps.

### Sleep Studies

Forty-eight participants who completed ventilatory chemoreflex measurements also completed sleep studies. Three participants were excluded due to unacceptable oximetry recordings, leaving a final cohort of 45 participants (Table 2). Participants were instrumented each night with a limited channel polysomnogram (Respironics Alice PDx, Murrysville, PA, United States). This recording included nasal pressure, finger pulse oximetry, thoracic and abdominal effort bands, electro-oculogram, two channel electroencephalogram, and chin electromyogram. Each subject was also fitted with a WatchPAT device, consisting of fingertip peripheral arterial tonometry and pulse oximetry (Itamar Medical, Caesarea, Israel), which was used in the event of an Alice PDx device failure. Studies were scored by a registered polysomnographic sleep technologist using American Academy of Sleep Medicine criteria for scoring and Chicago criteria for events (Berry et al., 2012).

**TABLE 2:** Demographics for participants with complete sleep study data.

Variable	Men (*n* = 22)	Women (*n* = 23)	*p*-value
Age (y)	48.6 ± 11.0	38.8 ± 13.8	0.011
BMI (kg/m^2^)	26.3 ± 3.4	27.8 ± 4.7	0.227
CMS Score	3.1 ± 3.8	0.6 ± 1.1	0.006
SBP (mmHg)	116.3 ± 14.4	107.0 ± 21.3	0.091
DBP (mmHg)	73.2 ± 10.5	72.2 ± 11.2	0.752
Hct (%)	56.6 ± 5.2	49.0 ± 5.7	<0.001
SpO_2_ (%)	84.6 ± 4.4	85.2 ± 4.6	0.651
PET_CO2_ (mmHg)	31.1 ± 4.5	32.6 ± 4.2	0.292
HR (bpm)	69.6 ± 9.5	74.3 ± 9.6	0.096

*Values listed as mean ± standard deviation and represent measures taken during wakefulness. *p*-values represent comparisons across sex groups.*

### Statistical Analysis

We tested the hypotheses that (1) ventilatory chemosensitivity (HVR and HCVR), and sleep disordered breathing [apnea-hypopnea index (AHI), obstructive apnea index (OAI), and/or desaturation events] would predict Hct and (2) individuals with higher Hct or CMS scores would have lower ventilatory chemosensitivity and increased measures of sleep disordered breathing. All statistical analyses were conducted in R Studio (R Studio, Inc.). Univariate associations and multivariate models were conducted in men and women independently due to the large effect of sex on Hct. Multivariate models were screened for collinearity and predictors were removed if they had a variance inflation factor (VIF) greater than 4 (O’Brien, 2007). Models for daytime variables in men were Hct or CMS Score ∼Age + BMI + SpO_2_ + HVR + PET_CO2_. Models for nighttime variables in men were Hct or CMS Score ∼Age + BMI + SpO_2_ + AHI + OAI. Models for women were Hct or CMS Score ∼Age + Menopause + BMI + SpO_2_ + HVR + PET_CO2_ and Hct or CMS Score ∼Age + Menopause + BMI + SpO_2_ + AHI + OAI, respectively. All variables in multivariate models were treated as continuous except menopause status (pre or post). Daytime SpO_2_ is the resting saturation measured during room air breathing. Nighttime SpO_2_ predictors were nadir SpO_2_, mean SpO_2_, or time spent below 80% SpO_2_. In the event of collinearity, the best SpO_2_ predictor based on highest *R*^2^ (nadir SpO_2_, mean SpO_2_, or time spent below 80% SpO_2_) was used in the final models. Values are presented as mean ± standard deviation throughout the manuscript.

## Results

### Low Daytime Saturation Is Associated With Hypoventilation and CMS Symptoms

**FIGURE 2:**
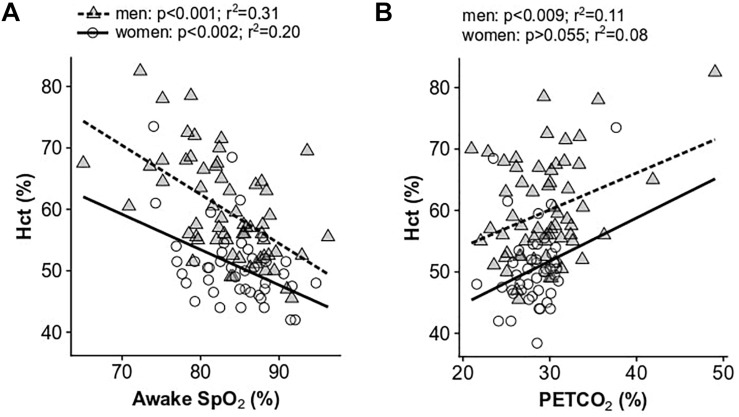
Univariate relationships between hematocrit and resting awake SpO_2_ **(A)** and resting end-tidal P_CO2_ **(B)**. Data for women are shown as open circles and solid lines, data for men are shown as gray triangles and dashed lines.

Low daytime SpO_2_ measurements were significantly correlated with increased Hct in both men and women (*p* < 0.001 and *p* < 0.002, respectively; Figure 2A) and with high CMS scores in men only (*p* < 0.001 and *r*^2^ = 0.12). HVR was not significantly associated with Hct nor CMS scores in men or women. Post-menopausal women had lower HVR (pre: 0.0009 ± 0.002 l/min/%SpO_2_/kg, post: 0.00006 ± 0.001 l/min/%SpO_2_/kg; *p* < 0.05) and lower resting SpO_2_ compared to their pre-menopausal counterparts (pre: 85.7 ± 4.5%, post: 82.2 ± 4.7%, *p* < 0.03). PET_CO2_, a measure of resting alveolar ventilation, correlated with both Hct and CMS score in men (*p* < 0.009 and *p* < 0.03, respectively) and showed a non-significant trend with Hct in women (*p* = 0.055; Figure 2B), although these relationships display substantial variation.

In multivariate models, resting daytime SpO_2_ was significantly associated with Hct in men (*p* < 0.0001), predicting 34% of the variance. In women, resting daytime SpO_2_ (*p* < 0.01), age (*p* < 0.006), and menopause status (*p* < 0.001) were associated with Hct, with the model predicting 43% of the variance. Daytime SpO_2_ was the only significant predictor of CMS scores in men (*p* < 0.02; 24% of the variance explained) and women (*p* < 0.05; 26% of the variance explained). There was no collinearity in the model predictors for Hct or CMS score, and all VIFs were less than 2. Results from multivariate models are provided in Table 3.

**TABLE 3:** Multiple regression models between Hct and CMS Score and daytime measures.

Daytime multivariate models							
Dependent variable	Sex	Independent variable	Coefficient of regression (β)	SE	*p*-value	Model *R*^2^	Model *p*-value
Hct	M	Age	–0.04	0.076	0.64	0.34	<0.001
		BMI	0.19	0.298	0.52		
		SpO_2_	–0.73	0.172	<0.001***		
		HVR	–260.96	258.678	0.32		
		PET_CO2_	0.19	0.226	0.41		
		Intercept	111.67	20.563	<0.001***		
	F	Age	0.29	0.098	0.005**	0.43	0.001
		Menopause	10.06	2.713	0.001**		
		BMI	–0.03	0.211	0.89		
		SpO_2_	–0.77	0.241	0.003**		
		HVR	100.75	585.644	0.86		
		PET_CO2_	–0.36	0.428	0.41		
		Intercept	108.66	29.614	0.001**		
CMS	M	Age	0.09	0.053	0.10	0.24	0.008
		BMI	0.04	0.208	0.86		
		SpO_2_	–0.31	0.120	<0.01*		
		HVR	–71.58	180.208	0.69		
		PET_CO2_	0.16	0.158	0.30		
		Intercept	21.15	14.325	0.15		
	F	Age	0.03	0.038	0.45	0.27	0.06
		Menopause	–1.36	1.039	0.20		
		BMI	0.05	0.081	0.51		
		SpO_2_	0.18	0.092	<0.05*		
		HVR	–13.84	224.347	0.95		
		PET_CO2_	0.31	0.164	0.07		
		Intercept	–24.34	11.344	<0.04*		

Asterisks indicate significant effects at the *p < 0.05, **p < 0.01, or ***p < 0.001 level.

### Sleep Desaturation and Sleep-Disordered Breathing Are Associated With Hematocrit and CMS Symptoms

**FIGURE 3:**
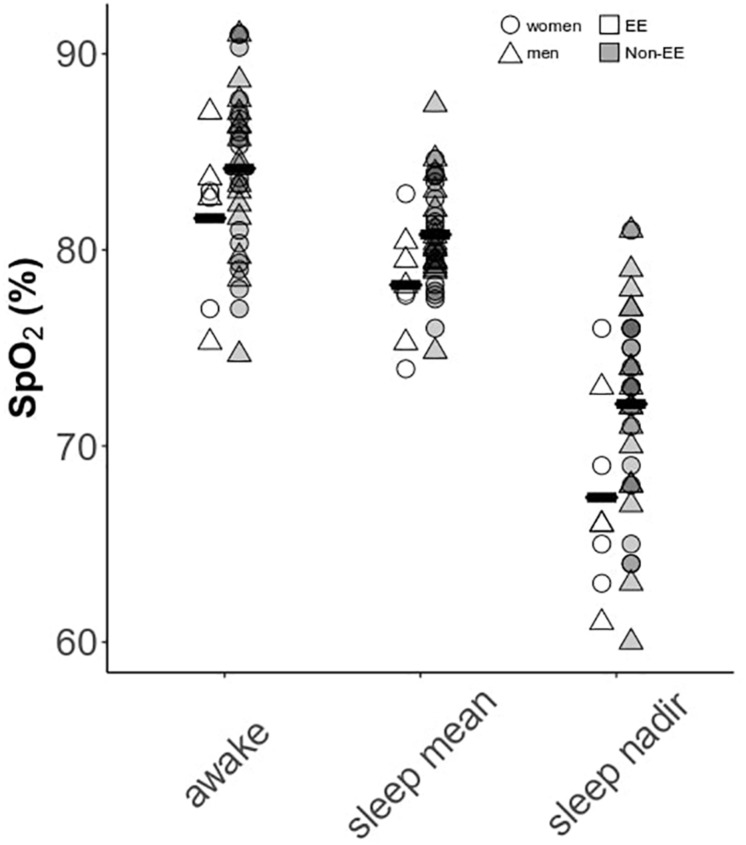
Awake versus sleep SpO_2_ parameters in men and women. Data are separated by the presence or absence of excessive erythrocytosis (EE). Means for each group are provided as solid black bars. *N* = 45 subjects who completed sleep studies.

**FIGURE 4:**
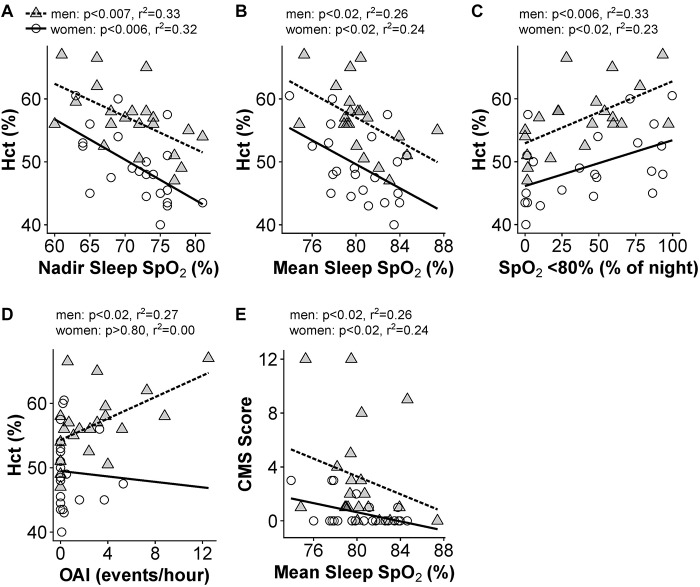
Significant univariate relationships between sleep variables and hematocrit **(A–D)** and CMS Score **(E)**. Data for women are shown as open circles and solid lines, data for men are shown as gray triangles and dashed lines. *N* = 45 subjects who completed sleep studies.

Men and women demonstrated substantially lower sleep mean and nadir SpO_2_ compared to awake values [Figure 3; *F*(2,84) = 190.5, *p* < 0.0001]: mean resting awake saturation (men: 84.6 ± 4.4; women: 85.2 ± 4.5%), mean sleep saturation (men: 80.3 ± 2.8; women: 80.3 ± 2.9%), and mean nadir sleep saturation (men: 71.0 ± 5.8; women: 71.5 ± 5.0%). Men and women with higher Hct had lower night-time mean and nadir SpO_2_ as well as greater time spent <80% SpO_2_ (Figures 4A–C). Participants with lower mean sleep SpO_2_ also had higher CMS scores (Figure 4E). No univariate association was detected between the AHI and Hct or CMS score. However, OAI was associated with Hct in men (Figure 4D), and there was a trend toward greater OAI and higher CMS score in men (*p* > 0.06).

In multivariate models, the three measures of night-time SpO_2_ (mean, nadir, and time below 80%) showed significant collinearity (VIF > 4). As a result, we tested which SpO_2_ measure provided the best predictive power in the Hct and CMS score models (Hct or CMS score ∼age + SpO_2_ + AHI + OAI). For Hct in men, nadir SpO_2_ and percent of the night below 80% SpO_2_ were similar (*R*^2^ = 0.58), while mean sleep SpO_2_ had slightly less predictive power (*R*^2^ = 0.53). The model including nadir sleep SpO_2_ gave the highest *F* value in both men [*F*(4,16) = 5.52, *p* < 0.01] and women [*F*(4,17) = 4.54, *p* < 0.05)], and nadir sleep O_2_ saturation was the only significant predictor of Hct in women (*p* < 0.02), while AHI and OAI were also significant predictors of Hct in men (*p* < 0.05 for both). For CMS Scores, mean sleep SpO_2_ gave the highest *F* value [*F*(4,16) = 3.4, *p* < 0.05, *R*^2^ = 0.46], and AHI and OAI also predicted CMS scores (*p* < 0.007 and *p* < 0.003, respectively) in men. The multivariate models did not produce any significant predictive power for CMS scores in women (*p* > 0.05 regardless of O_2_ saturation measure included). All VIFs in these multivariate models were 3.2 or lower. Table 4 provides results for multivariate models of sleep parameters.

**TABLE 4:** Multiple regression models between Hct and CMS Score and sleep measures.

Sleep Multivariate Models							
Dependent variable	Sex	Independent variable	Coefficient of regression (β)	SE	*p*-value	Model *R*^2^	Model *p*-value
Hct	M	Age	–0.10	0.111	0.40	0.58	0.015
		BMI	–0.02	0.367	0.96		
		Nadir Sleep Desaturation	–0.36	0.241	0.15		
		AHI	–0.19	0.080	0.03*		
		OAI	1.31	0.476	0.02*		
		Intercept	88.42	27.949	0.006**		
	F	Age	0.22	0.110	0.06	0.59	0.021
		Menopause	4.17	3.051	0.19		
		BMI	0.21	0.239	0.40		
		Nadir Sleep Desaturation	–0.62	0.223	0.01*		
		AHI	–0.01	0.127	0.92		
		OAI	–1.24	1.222	0.33		
		Intercept	77.99	19.653	0.001**		
CMS	M	Age	0.10	0.092	0.29	0.51	0.039
		BMI	0.36	0.276	0.21		
		Mean Sleep SpO_2_	0.58	0.407	0.17		
		AHI	–0.21	0.065	0.007**		
		OAI	1.41	0.391	0.003**		
		Intercept	–57.63	40.329	0.17		
	F	Age	–0.01	0.029	0.74	0.24	0.599
		Menopause	–0.93	0.812	0.27		
		BMI	0.01	0.060	0.85		
		Mean Sleep SpO_2_	0.01	0.007	0.28		
		AHI	0.02	0.034	0.64		
		OAI	–0.22	0.319	0.50		
		Intercept	0.84	2.002	0.68		

Asterisks indicate significant effects at the *p < 0.05 or **p < 0.01 level.

### Ventilatory Chemoreflexes Are Associated With Daytime and Sleep O_2_ Saturation

**FIGURE 5:**
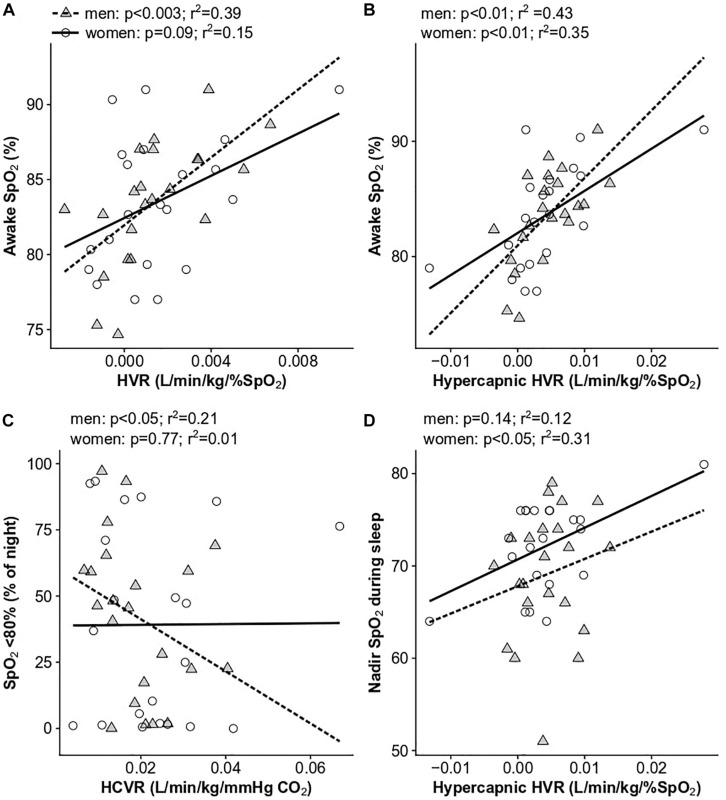
Significant univariate relationships between ventilatory chemoreflexes and SpO_2_ parameters: **(A)** HVR and awake SpO_2_, **(B)** hypercapnic HVR and awake SpO_2_, **(C)** HCVR and the percent of the night spent below 80% SpO_2_, and **(D)** hypercapnic HVR and nadir sleep SpO_2_. Data for women are shown as open circles and solid lines, data for men are shown as gray triangles and dashed lines. *N* = 45 subjects who completed sleep studies.

Our hypothesis that ventilatory chemoreflexes would predict Hct was not supported (Table 3). However, to determine if ventilatory chemoreflexes were associated with day or night-time SpO_2_, we examined the relationship of all SpO_2_ and sleep variables with the HVR, HCVR, and hypercapnic HVR in the sleep study cohort. Men with high HVRs maintained high mean daytime SpO_2_ (*r*^2^ = 0.39, *p* < 0.003; Figure 5A), and elevated hypercapnic HVR was associated with increased SpO_2_ in both men (*r*^2^ = 0.37, *p* < 0.01) and women (*r*^2^ = 0.35, *p* < 0.01; Figure 5B). The time spent below 80% SpO_2_ during sleep was significantly associated with the hypercapnic HVR (*r*^2^ = 0.25, *p* < 0.05) and HCVR (*r*^2^ = 0.21, *p* < 0.05) in men and showed a non-significant trend with HVR in women (*r*^2^ = 0.19, *p* < 0.06) (Figure 5C). In women, the nadir sleep O_2_ saturation was associated with the hypercapnic HVR as well (*r*^2^ = 0.31, *p* < 0.01; Figure 5D). No measures of ventilatory sensitivity were associated with the OAI or AHI.

### Men With Higher Hematocrit Have Lower Heart Rate Responses to Hypoxia

**FIGURE 6:**
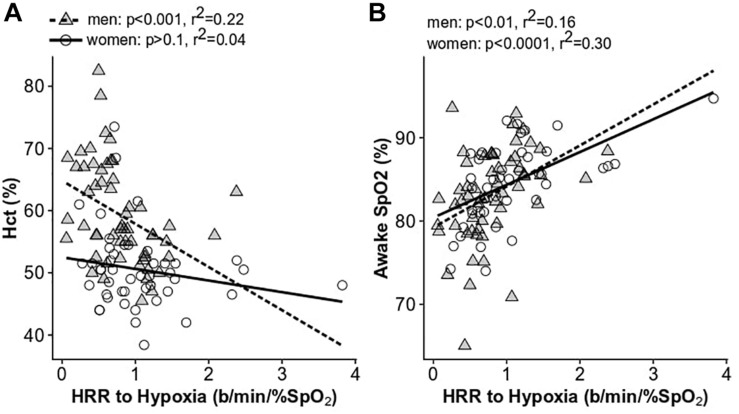
The relationship between the heart rate response to hypoxia and hematocrit **(A)** and daytime SpO_2_ **(B)**. Women are shown as open circles and solid lines; men are shown as gray triangles and dashed lines.

Men with lower Hct had larger increases in the HRR to acute hypoxia, while men with high Hct had very low HRR to hypoxia, or none (*p* < 0.001, *r*^2^ = 0.22; Figure 6A). A similar trend was observed in women (Figure 6), although the difference was not significant. These results were upheld when correcting for an interactive effect with age (men: *p* < 0.005; women: *p* < 0.5). The HRR to hypoxia decreased significantly with age in women (*p* < 0.008, *r*^2^ = 0.15) and was lower in post-menopausal women (post: 0.74 ± 0.45 beats/min/%SpO_2_, pre: 1.20 ± 0.70 beats/min/%SpO_2_; *p* < 0.01). The HRR to hypoxia was also associated with daytime SpO_2_ in both men and women (men: *p* < 0.01, *r*^2^ = 0.16, women: *p* < 0.0001, *r*^2^ = 0.30; Figure 6B). In contrast to the HRR to hypoxia, there was no relationship between the HRR to CO_2_ and Hct or SpO_2_ in men or women (*p* > 0.1 for all comparisons in men and women). Neither the HRR to hypoxia nor CO_2_ were associated with AHI, OAI, or sleep SpO_2_ measures in men or women before or after correcting for age.

## Discussion

### Ventilation

While HVR was not directly associated with Hct as we predicted, it was associated with daytime saturation in both men and women (Figures 5A,B), indicating blunted HVR may play a role in exacerbating hypoxemia and the development of excessive erythrocytosis. Furthermore, the increased PET_CO2_ in individuals with high Hct may indicate a decrease in baseline ventilatory drive, further exacerbating hypoxemia in this group. These results are in agreement with previous studies demonstrating higher PET_CO2_ values in high-altitude residents with CMS compared to those without CMS, but no differences, or very modest differences, across these groups in the acute HVR (Severinghaus et al., 1966; Lahiri et al., 1983; Fatemian et al., 2003).

In multivariate models, daytime SpO_2_ was the only significant predictor of Hct or CMS score in men. Since SpO_2_ is so strongly associated with Hct, it is not surprising that higher CMS scores are associated with lower SpO_2_ levels. In women, Hct was predicted by SpO_2_, age, and menopausal status (Table 3). CMS is more common after menopause (León-Velarde et al., 1997, 2001), which is consistent with the higher CMS scores reported by older women in this cohort and higher Hct values in older and post-menopausal women.

### Sleep

All SpO_2_ measures taken during sleep were associated with Hct in men and women (Figures 4A–C), which supports previous findings regarding mean and time spent below 80% SpO_2_ in this population (Spicuzza et al., 2004; Villafuerte et al., 2016; Pham et al., 2017b). Mean sleep SpO_2_ was also associated with CMS score in men only (Figure 4E). The severe desaturation events during sleep may explain why Villafuerte et al. (2016) showed serum Epo during sleep was significantly higher in men with CMS, in contrast to findings of similar Epo levels in CMS and healthy male participants during the day (León-Velarde et al., 1991; Villafuerte et al., 2014; Hsieh et al., 2016). Villafuerte et al. (2016) also showed that mean sleep SpO_2_, total sleep time below 80% SpO_2_, and the Epo-to-soluble Epo receptor ratio (an Epo availability index) were significant predictors of Hct in men. Finally, Julian et al. (2013) found that men with excessive erythrocytosis had lower nocturnal SpO_2_ as well as higher levels of the oxidative stress marker 8-iso-PGF2alpha. The results presented here demonstrate that the relationships between sleep SpO_2_ and Hct previously identified in men are present in women as well.

AHI did not display a univariate association with Hct in men or women. This result supports the findings of Spicuzza et al. (2004) and Pham et al. (2017b) who report no difference in AHI across men with or without excessive erythrocytosis, but contrasts with Julian et al. (2013) who found higher AHI during REM sleep in men with excessive erythrocytosis. In contrast, OAI was positively associated with Hct in men (Figure 4D), which differs from the results reported by Spicuzza et al. (2004) who found only one excessive erythrocytosis participant who displayed obstructive apneas among the 10 CMS and 10 non-CMS participants examined; this difference may be attributed to differences in sample size or sampling method and the fact that the average age of our male participants was 10 years greater than that of Spicuzza et al. (2004). However, we also conducted multivariate models which controlled for age to determine the best predictors of Hct and CMS score. In these models, OAI and AHI were both predictors of Hct and CMS score in men but not women.

While we did not find that differences in chemosensitivity accounted for the presence or absence of sleep disordered breathing, control of breathing clearly influences factors such as respiratory event duration and arousal from sleep. Unlike Spicuzza et al. (2004), we did not see a significant relationship between the HCVR and AHI, but men with lower HCVR did spend more time below 80% SpO_2_ at night (Figure 5C). The hypercapnic HVR was associated with nadir SpO_2_ during sleep in women (Figure 5D), but not men, indicating sex-specific differences may underlie distinct outcomes that could be attributed to prolonged versus frequent desaturation events. All this considered, it is possible that low ventilatory drive contributes to larger reductions in nighttime SpO_2_ and exacerbates erythropoiesis and/or other commonly associated outcomes. Metrics to characterize patterns of hypoxemia (e.g., prolonged versus frequent desaturation events) (Azarbarzin et al., 2019) may be useful to better understand which individuals are at highest risk of excessive erythrocytosis and CMS.

Several studies have shown that intermittent hypoxia resulting from sleep apnea is associated with systemic hypertension, coronary artery disease, heart failure, and metabolic dysfunction (Jean-Louis et al., 2008; Badran et al., 2014; Dewan et al., 2015; Floras, 2015; Torres et al., 2015; Ryan, 2017, 2018). Therefore, patterns of hypoxia during sleep at high altitude likely contribute to the poor cardiometabolic outcomes observed in Andeans (Pham et al., 2017b). Efforts to assess further the molecular effects of intermittent hypoxemia during sleep, on top of continuous chronic hypoxia in this population (Prabhakar and Semenza, 2012), will provide important clues into the development of CMS and related cardiometabolic pathologies. Longitudinal studies that examine the progression of excessive erythrocytosis and CMS are needed to address better the cascade of events underlying these outcomes in this population.

### Heart Rate Response to Hypoxia

We also found that Hct was associated with the HRR to hypoxia in men (Figure 6). Men with excessive erythrocytosis have a lower HRR to acute hypoxia after correcting for age (*p* < 0.05) (Kronenberg and Drage, 1973). This provides further evidence of blunted chemoreceptor sensitivity or autonomic outflow in this group. Previous work has also demonstrated a lower HRR to orthostasis in CMS patients compared to non-CMS high-altitude residents despite exceptional orthostatic tolerance in both groups (Claydon et al., 2004a). CMS patients also have lower reflex vasoconstriction and impaired cerebral blood flow autoregulation at sea level compared to non-CMS highlanders (Claydon et al., 2004b). It was suggested that these responses may result from a higher “set point” of the carotid baroreceptor-vascular resistance reflex (Moore et al., 2006). Lower carotid chemoreceptor and baroreceptor function may be a compensatory response to chronically elevated sympathetic activity resulting from chronic hypoxemia. It may also be the typical response to chronic hypoxia exposure as previous evidence suggests blunted hypoxic ventilatory chemosensitivity in sojourners as a function of time spent at high-altitude (Zhuang et al., 1993). In either case, loss of chemoreceptor function puts individuals with excessive erythrocytosis at higher risk of severe desaturation during exertion or apneic periods during sleep.

## Conclusion

We demonstrate that lower SpO_2_ indices during sleep and during the day are associated with higher Hct in Andean men and, for the first time, in women. When controlling for age and SpO_2_, OAI and AHI also predicted Hct and CMS scores in men. While the HVR is blunted in Andeans with and without excessive erythrocytosis, lower hypoxic chemosensitivity was associated with lower daytime SpO_2_ and may therefore play a role in excessive erythrocytosis development. While these results support previous work in this population, we are the first to provide paired sleep and ventilatory chemoreflex measurements in the same Andean men and women, and the first to show that the HRR to hypoxia is also blunted in excessive erythrocytosis in men. A limitation of this study is that we cannot determine if more severe hypoxemia, or other phenotypes associated with Hct, are a cause or effect of excessive erythrocytosis. Interventional and longitudinal studies are required to determine the causal relationships between these associations.

## Data Availability Statement

The raw data supporting the conclusions of this article will be made available by the authors, without undue reservation, to any qualified researcher.

## Ethics Statement

This study was conducted in accordance with the Declaration of Helsinki, except for registration in a database, and was approved by the University of California, San Diego Human Research Protection Program. Participants provided written consent in their native language (Spanish).

## Author Contributions

TS and FV designed and oversaw the study. EH, JO, DG, CA-R, PD, MD, NC, GV-G, JM, EG, and FP participated in participant screening and data collection. Sleep scoring was performed by PD and data analyses were performed by EH, AM, and JO. EH, JO, TS, and FV produced the original draft and all authors contributed to the final manuscript.

## Conflict of Interest

The authors declare that the research was conducted in the absence of any commercial or financial relationships that could be construed as a potential conflict of interest.
